# The Contribution of Functional Traits to the Breeding Progress of Central-European Winter Wheat Under Differing Crop Management Intensities

**DOI:** 10.3389/fpls.2019.01521

**Published:** 2019-12-06

**Authors:** Till Rose, Henning Kage

**Affiliations:** Institute of Crop Science and Plant Breeding, Agronomy and Crop Science, Christian-Albrechts-University, Kiel, Germany

**Keywords:** wheat, breeding progress, harvest index, biomass, radiation interception, radiation use efficiency, high-throughput phenotyping, uav

## Abstract

Wheat yields in many of the main producing European countries stagnate since about 20 years. Hence, it is of high interest, to analyze breeding progress in terms of yield and how associated traits changed. Therefore, a set of 42 cultivars (released between 1966 and 2012) was selected and yield as well as functional traits defined by the Monteith and Moss equation were evaluated under three levels of management intensity. The Monteith Moss equation thereby calculates grain yield as the product of incident photosynthetically active radiation, fraction of intercepted radiation, radiation use efficiency, and harvest index. The field trial was performed in a high yielding environment in Northern Germany in two seasons (2016–2017 and 2017–2018) with very contrasting rainfall rates. The three differing managements were: intensive (high N + pesticides), semi-intensive (high N − pesticides), and extensive (low N − pesticides). The results indicate that the stagnation of wheat yields in Central-Europe is not caused by a diminishing effect of breeding on yield potential. This equally applies to suboptimal growing conditions like extensified crop management and restricted water supply. Nearly all functional sub-traits showed a parallel progress but coefficients of determination of relationships between traits and year of variety release are decreasing along the hierarchy of yield formation. One exception is radiation interception which did not show a stable linear increase during breeding history. In recent years, biomass is getting more important in comparison to harvest index. Values of harvest index are slowly approaching theoretical maxima and correlations with grain yield are decreasing.

## Introduction

Breeding progress of common wheat (*Triticum aestivum* L.) recently gained much attention because after at least half a century of continuing increase of farm level wheat yields (Calderini and Slafer, 1998) this process stagnates in many of the main producing countries, including France, the United Kingdom, and Germany (Lin and Huybers, 2012). These countries achieve above-average yields and therefore have major importance for worldwide wheat supply. An analysis of the breeding progress of the past can help to get back on track for a much-needed increase of wheat yields.

A deeper understanding if yield formation was analyzed using the equation (1) (Monteith and Moss, 1977):

(1) $$\mathrm{GY}=\overset{\text{harvest}}{\underset{\text{sowing}}{\sum }}{\left[R_{\mathrm{PAR}}\times \mathrm{RI}\times \mathrm{RUE}\right]}_{\mathrm{BIO}}\times \mathrm{HI},$$

which calculates grain yield (*GY*) as the product of incident photosynthetically active radiation (*R*_PAR_), fraction of intercepted radiation (*RI*), radiation use efficiency (*RUE*), and harvest index (*HI*). This equation can be aggregated to the term: *GY* is the product of above-ground biomass (*BIO*) and *HI*.

The amount of incident radiation is determined by the location, its seasonal weather, and the dates of sowing and harvest. The intercepted fraction of this radiation is defined by the speed of development, the final size, the longevity, and the architecture of the canopy (Long et al., 2006). A high speed of canopy development is labeled “early-vigor”. Under Central-European growing conditions losses until canopy closure mainly occur during March and April, when the green area index (*GAI*) of the canopy is not sufficient for near total interception and in parallel usable incoming radiation occurs (Rose et al., 2017). A fast canopy development in this early phase can increase biomass production.

The longevity of the canopy mainly depends on its senescence dynamics. When canopies reach their final size the subsequent phase of maximal photosynthesis is rapidly replaced by the phase of senescence. Here, physiological integrity is maintained in the beginning but complete self-destruction finally terminates all growth processes (Thomas and Smart, 1993). Like the subtle process of canopy formation, its senescence is a well-programmed sequence and after the period of carbon assimilation, leaves now contribute to the grain yield by the remobilization of their nutrients (Wu et al., 2012). The visible symptom of leaf senescence is the loss of chlorophyll and genotypes which express delayed chlorophyll catabolism are named “stay-green” (Thomas and Ougham, 2014). Functional “stay-green” genotypes maintain carbon assimilation for a prolonged period during grain-filling (Thomas and Howarth, 2000; Rebetzke et al., 2016), this is often associated with considerable yield differences in wheat trials (Verma et al., 2004; Luo et al., 2006; Kichey et al., 2007; Christopher et al., 2008; Wang et al., 2008; Bogard et al., 2011; Wu et al., 2012; Christopher et al., 2016; Montazeaud et al., 2016; Pinto et al., 2016; Luo et al., 2018). In contrast, unfavorably delayed leaf senescence might result in a low nitrogen use efficiency as well as a low grain protein content (Wu et al., 2012). A tradeoff between a longer maintenance of leaf chlorophyll and a less efficient remobilization of nitrogen has been shown by Gaju et al. (2011). Therefore, Thomas and Ougham (2014) define the ideotype to “comprise late initiation of canopy senescence, to maximize C capture, followed by fast and complete mobilization of N and other nutrients”.

The onset of senescence is both, part of the development process of a plant and inducible by numerous external factors (temperature, drought, nutrient supply, pathogen attack) (Buchanan-Wollaston, 1997). The developmental senescence is a consequence of phenology and induced by internal signals such as phytohormones. Stress-induced senescence in contrast is triggered by external signals (Derkx et al., 2012). These signals are primarily nitrogen depletion (Osaki et al., 1991; Mi et al., 2000; Derkx et al., 2010; Bogard et al., 2011; Luo et al., 2018), water depletion (Idso et al., 1980; Christopher et al., 2008; Thomas and Ougham, 2014; Christopher et al., 2016; Christopher et al., 2018), and pathogens (Berdugo et al., 2013). So, a “stay-green” phenotype can represent the phenological component per se but also just reflects the ability to cope with the aforementioned external factors (Richards, 2000; Rebetzke et al., 2016).

The amount of intercepted radiation is multiplied by the radiation use efficiency to get the final biomass, the measure for biological performance. This transformation efficiency includes all forms of stress (drought, pathogens, etc.) but is also affected by crop architecture (Zhu et al., 2010) and light saturated photosynthetic rates (Gaju et al., 2016). The increase of radiation use efficiency in a historical breeding set was not related to photosynthesis but shifts in canopy-level traits (Sadras et al., 2012).

The final biomass is multiplied with harvest index to get the grain yield, the measure for agronomical performance. In contrast to the preceding steps of yield formation which can be improved by the optimization of manifold biological processes, here, the quite simple parameter—fraction of biomass that is part of the harvested organs—can considerably change the final grain yield. The upper border of this fraction is restricted by an increased risk of lodging (Berry et al., 2007) and eventually occurring negative interactions with the processes of biomass production. Educated guesses for this threshold are: 0.62 (Austin, 1980), 0.64 (Foulkes et al., 2010), and 0.66 (Shearman et al., 2005).

For an adequate representation of environmental variables which influence complex traits, uncontrolled field trials are still the only adequate facility. Aerial platforms—especially unmanned drones—are an attracting platform for the use in these large-scale open field trials. Equipped with different sensors, they perform high throughput phenotyping with a high spatial resolution (Haghighattalab et al., 2016; Pauli et al., 2016; Araus et al., 2018; Condorelli et al., 2018). Additionally, in comparison to ground based methods, airborne methods often reach a higher precision because they can represent the whole plot (statistical selection can be applied to all pixels) and confounding environmental effects (temperature, sun angle etc.) are reduced through the short measurement time (Tattaris et al., 2016). This can result in higher repeatabilities for a trait like the NDVI (Condorelli et al., 2018).

The progress in the development of small unmanned drones, in combination with calibrated spectral sensors for the prediction of whole season green area indices, now allows to measure all components of the yield equation by Monteith and Moss (1977) in large field trials.

The aim of this study is to analyze the functional pathways historical breeding used to improve yield potential of winter wheat in Central-Europe. To enlighten the black box of the preceding breeding success can help to open new perspectives for the contribution of breeding to a much-needed increase of wheat yields. The analysis is complemented by suboptimal crop management intensities and experimental years with contrasting water supply which might become more relevant due to environmental concerns and a changing climate.

## Material and Methods

### Field Trials

The field trials were conducted during two consecutive years (growing seasons 2016–2017 and 2017–2018). Sowing date was from September 20 to September 22, 2016 and on October 17, 2017. Harvest date was from August 8 to August 15, 2017 and on July 27, 2018 (**Table 1**). The experiment comprised two factors: crop management and winter wheat cultivar, and the design was laid out as a split-plot with three replications in which the factor crop management was nested within replication and the factor cultivar was nested within crop management.

**Table 1:** Main agronomical and phenological dates during both growing seasons.

	Season 2016–2017	Season 2017–2018
Sowing date	Sep. 21 (Sep. 20–Sep. 22), 2016	Oct. 17, 2017
Ear emergence complete	Jun. 3 (May 28–Jun. 8), 2017	Jun. 1 (May 29–Jun. 06), 2018
Hard dough	Jul. 23 (Jul. 19–Aug. 1), 2017	Jul. 14 (Jul. 09–Jul. 18), 2018
Harvest date	Aug. 11 (Aug. 8–Aug. 15), 2017	Jul. 27, 2018

Values in parentheses indicate the range of the analyzed set.

The factor cultivar includes 42 levels. The set of cultivars is chosen to represent the German breeding progress in the period from 1966 to 2012 (for more details see **Table 2**). The factor crop management includes three levels: intensive, semi-intensive, and extensive. The intensive treatment received mineral fertilizer at a total N supply rate of 220 kg N ha^−1^ (fertilization adjusted for soil mineral nitrogen, N_min_) as well as full intensity of fungicides, insecticides, and growth regulators, representing standard wheat production systems in Central-Europe. The semi-intensive treatment received mineral fertilizer at the same level as well as growth regulators, but no fungicides and insecticides, representing a scenario with no chemical plant protection. The extensive treatment received mineral fertilizer at a total N supply rate of 110 kg N ha^−1^ (fertilization adjusted for N_min_), no fungicides and insecticides, and no growth regulators in the first season (due to problems with lodging growth regulators were applied in the second season), representing a scenario of comprehensive extensification in crop production.

**Table 2:** Detailed information about the set of analyzed cultivars.

Cultivar	Year of release	Breeder	Quality group
Diplomat	1966	Firlbeck	A
Caribo	1968	Heidenreich und Eger	B
Highbury	1968	Plant Breeding International Cambridge (PBIC)	A
Progress	1969	Hege	A
Kormoran	1973	Lochow-Petkus	A
Disponent	1975	Bayrische Saatzuchtgesellschaft	A
Monopol	1975	Firlbeck	E
Carimulti	1975	Heidenreich und Eger	C
Vuka	1975	Franck	A
Götz	1978	Bayrische Saatzuchtgesellschaft	B
Tabor	1979	Strube	A
Kronjuwel	1980	Bayrische Saatzuchtgesellschaft	B
Urban	1980	Bauer, G.	E
Rektor	1980	Firlbeck	E
Kanzler	1980	Engelen Büchling	B
Sperber	1982	Lochow-Petkus	A
Ares	1983	Strube, Dr. H.	B
Apollo	1984	Saatzucht Breun	C
Obelisk	1987	Strube, Dr. H.	B
Alidos	1987	Saatzucht Hadmersleben	E
Greif	1989	Lochow-Petkus	B
Bussard	1990	Lochow-Petkus	E
Kontrast	1990	Saatzucht Hadmersleben	A
Ritmo	1993	Cebeco	B
Batis	1994	Strube, Dr. H.	A
Pegassos	1994	Strube Saatzucht	A
Flair	1996	Schweiger	B
Cardos	1998	Saatzucht Hadmersleben	A
Dekan	1999	Lochow-Petkus	B
Drifter	1999	Nickerson	B
Biscay	2000	Lochow-Petkus	C
Tommi	2002	Nordsaat	A
Akteur	2003	DSV	E
Hermann	2004	Limagrain-Nickerson	C
Türkis	2004	Lantmännen SW Seed Hadmersleben GmbH	A
Potenzial	2006	DSV	A
Manager	2006	Saatzucht Schweiger GbR	B
Inspiration	2007	Saatzucht Josef Breun GmbH & Co.KG	B
JB Asano	2008	Saatzucht Josef Breun GmbH & Co.KG	A
Tobak	2011	W. v. Borries-Eckendorf	B
Patras	2012	DSV	A
Elixer	2012	W. v. Borris-Eckendorf	C

Year of cultivar release, breeder, and baking quality group. German baking quality groups are: E (elite, premium quality bread wheat), A (bread wheat), B (milling wheat), and C (feed quality).

To prevent patchy effects from weed cover, chemical control measures were applied across all crop management treatments (including extensive). Nutrients other than N were applied consistent across all crop managements according to requirements determined individually in each year.

To avoid neighboring effects between plots (caused by differing plant heights) and to allow separate harvest of early and late maturing cultivars, those were grouped by expected plant height and maturation time in four incomplete sub-blocks (early/short, early/high, late/short, late/high) which were randomized within crop management. Those sub-blocks were omitted in the second season because it has shown that differing maturation time is not a problem (all sub-blocks were harvested in parallel) and plant height is quite similar when growth regulators are applied.

The experiments were part of the project BRIWECS (breeding innovations wheat for resilient cropping systems). An overview analysis for context can be seen in Voss-Fels et al. (2019).

### Site, Soil, and Weather

The field trials were conducted at the Hohenschulen Experimental Farm (northern Germany, 54°18′51.2″ N 9°59′28.8″ E, 30 m a.s.l). The soil is characterized as a pseudogleyic sandy loam (Luvisol: 170 g kg^−1^ clay, pH 6.7, 13 g kg^−1^ C_org_, 1.1 g kg^−1^ N_org_ in 0–30 cm). The climate of northern Germany is humid temperate with a long-term mean annual temperature at the location of 8.8°C and mean annual precipitation of 751 mm, whereof 396 mm occur during the main growing season (March–August).

Daily weather was recorded at a station nearby the field trials. In 2016–2017 temperatures below average occurred from the beginning of October to the mid of February with the exception of a warm period during December. A cold period appeared again from mid of April to the mid of May. After a period with low precipitation from November to March, the season was characterized by high precipitation from March until harvest. Levels of global radiation were on average.

In the season 2017–2018 temperatures above average occurred almost during the whole season with the exception of a cold period in February and March. After a period with altering phases of below and above precipitation until the end of April, a long period of very low precipitation until harvest followed. Levels of global radiation were above average in May and July.

In summary, the seasons mainly differed regarding the amount of precipitation after anthesis (**Table 3**) and the phenological development during grain filling as a result of air temperatures and drought (**Table 1**). Weather in comparison to climate during both growing seasons is shown in more detail in **Figure S1**.

**Table 3:** The Amount and distribution of precipitation as well as effective PA-radiation during the growing seasons.

	2016–2017		2017–2018	
	Before anthesis	After anthesis	Before anthesis	After anthesis
Precipitation [mm]	400	198	356	24
PAR_effective_ [MJ m^−2^ d^−1^]	595	442	599	432

### Measurements and Calculations

#### Grain Yield, Biomass, Harvest Index, and Phenology

All plots were harvested by combine between August 8 and August 15, 2017 and on July 27, 2018, respectively. Grain yield was standardized to 100% dry matter. In addition, harvest by hand was performed at 0.5 m along the row in the first season (corresponds to 0.06 m^2^) and 1 m along the row in the second season (corresponds to 0.12 m^2^) to ascertain the harvest index (ratio of grain dry matter to total dry matter). Harvest by hand took place 6–13 days before combine harvest in 2017 and 4 days before combine harvest in 2018.

Small samples, like the hand harvest, are adequate to measure ratios between fractions but not absolute values. These are appreciable affected by variation inside a plot. As a consequence, we calculated biomass as the ratio of grain yield (combine) and harvest index (hand harvest) to minimize sampling errors.

Lodging and/or damage by game animals occurred in the 2017 season. Plots were visually classified and damaged ones were excluded from the analysis (12%). In the season 2018 no disturbance occurred.

For the detailed analysis of drought, the variables grain yield drop (*GY drop*) and biomass drop (*BIO drop*) are introduced. These are calculated as the difference between the stressed season (2018) and the unstressed season (2017). Consequently, the higher the effect of drought the lower the value (usually more negative).

Phenological stages ear emergence and hard dough were visually classified in the intensive crop management by frequent observations in the relevant periods. These stages refer to the states 59 and 87, respectively, in the BBCH-scale (Lancashire et al., 1991). For statistical analysis both traits were transformed to growing degree days (base temperature 0°C) to improve comparison between both seasons.

#### Radiation Interception and Radiation Use Efficiency

The amount of intercepted radiation can be described following Beer–Lambert law (Monsi and Saeki, 1953):

(2) $$I=I_{0}\times \left(1-e^{-k\times \mathrm{GAI}}\right)$$

where *I*_0_ is the incoming radiation, *k* the extinction coefficient and *GAI* the one-sided area of all green plant material per ground area. To focus on interception of productive radiation, the incoming radiation was weighted by a temperature weighting factor as a function of the daily mean temperature. The function ranges between 0 and 1 and is trapezoidal with transition points at 2.5°C, 9.5°C, 20°C, and 35°C. As a consequence, *I* represents the intercepted effective radiation. The extinction coefficient *k* is ascertained to be 0.7 (unpublished own measurements) for the whole genotype set. Differences regarding *k* might occur but are assumed to be of minor importance for radiation interception (sensitivity of *k* to differing leaf angles is quite low around 45° sun angle, where most radiation occurs at the latitude of the location). Values of *GAI* were determined for every single plot with a high measurement frequency (at least biweekly during the main growing phase, in total 11,538 data points, **Table 4**) to provide suitable interpolated values for every single day. Calculation of total incoming effective radiation and total intercepted effective radiation starts with the mean day of sowing and ends with the mean day of the phenological stage hard dough in each season. This represents the period of land use by the analyzed crop. The ratio of total intercepted effective radiation to total effective radiation is hereafter mentioned as fraction of intercepted radiation (*RI*). Radiation use efficiency (*RUE*) was calculated as the ratio of final above-ground biomass to the sum of intercepted effective radiation so the parameter describes the potential *RUE* (when temperature is optimal throughout the whole season).

**Table 4:** Date and method of *GAI* measurements.

	Season 2016–2017	Season 2017–2018
Ortho images	Dec. 28, Mar. 9	–
Reflection measurements	Apr. 03, Apr. 19, May 7, May 15, May 23, May 29, Jun. 14, Jun. 19, Jun. 27, Jul. 3, Jul. 13	Dec. 4, Mar. 9, Apr. 9, Apr. 16, Apr. 23, May 3, May 16, May 22, Jun. 1, Jun. 6, Jun. 12, Jun. 20, Jun. 26, Jul. 5,. Jul. 13, Jul. 17

##### Green Area Index

Measurements of *GAI* were conducted by two different methods: ortho images and reflection measurements. In the beginning of the first season *GAI* values were tracked using the ortho image method, all subsequent dates were measured using the reflection measurement method. In total, 13 measurement dates exist in the season 2016–2017 and 16 measurement dates exist in the season 2017–2018.

For the calculation of radiation interception, daily values of *GAI* are necessary. For interpolation, locally weighted scatterplot smoothing (LOESS) (Cleveland, 1979) was applied as proposed by Magney et al. (2016) instead of more functional growth curves (e.g. sigmoidal) because events like initiating drought could not be represented adequately by quite rigid growth curves. As LOESS algorithm, the homonymous function in base *R* (R Core Team, 2018) was used. The smoothing parameter α was set to 0.5 in the season 2016–2017 and 0.45 in the season 2017–2018.

Ortho images are RGB photos taken approximately 1.5 m above the ground with a view direction perpendicular to the grounds surface. All pixels are classified in plant as well as ground pixels and the ratio from plant pixels to the total number of pixels (ground cover) closely correlates with the *GAI*. Ortho-images were not taken in every plot because of restricted throughput using this ground-based method. On December 28, 2016 one image for every cultivar and on March 10, 2017 one image for every cultivar management combination was taken.

The RGB images were cropped to reduce angular effects at the border area and each pixel was classified into the groups plant and ground by a support vector machine with linear kernel using the R-package *e107* (Meyer et al., 2017) streamlined by the R-package *caret* (Kuhn, 2017). The algorithm was trained by manually classified pixels (16,629 in total, 99 different images), in doing so 80% of the images were used as training set and 20% set aside as test set. Chosen predictor variables are: red, green, blue, mean red value of the whole image, mean green value of the whole image, and mean blue value of the whole image. Predictors are centered and scaled. The tuning parameter *cost* was set to 0.04 using the largest value in a grid search with 10-fold cross validation. The trained support vector machine is reliably able to differentiate between plant and soil pixels, reaching a sensitivity of 0.88 and a specificity of 0.90 in the test set. Ground cover was calculated for each image as the ratio of plant pixels to total pixels.

Ground cover values were transformed into *GAI* values using the equation 3,

(3) $$\mathrm{GAI}=-\frac{\log\left(T\right)}{k},$$

whereof *T* (transmission) corresponds to 1 − ground cover and *k* (extinction coefficient) is assumed to be 0.75 on 28 December 2016 and 0.65 on 10 March 2017 to account for the erecting of leaves during development.

Reflection measurements were conducted with the Sequoia camera (Parrot), a multispectral sensor which records simultaneously images in four wavebands: green (550 nm), red (660 nm), red edge (735 nm), and near-infrared (790 nm). Red edge has a bandwidth of 10 nm, all other wavebands have a bandwidth of 40 nm. The Sequoia camera has an incoming light sensor and therefore provides fractional reflection values regarding the incoming radiation. On each measurement date, images of a grayscale target were made for radiometric calibration.

The eBee Plus (senseFly) served as carrier system. It is a lightweight fixed-wing drone operated with the flight manager eMotion 3 (senseFly). The chosen resolution was 8 × 8 cm pixel^−1^ and a high degree of overlap (80% in both directions) was applied which resulted in adequate raw material for post-processing with the photogrammetry software Pix4Dmapper (Pix4D SA., Switzerland). On days with fast-moving clouds, a manual screening of the images was conducted prior post-processing to exclude those images containing both, regions with cloud shadow and full sunlight. The results were four orthogonal reflection maps, one for every waveband. With a RTK-enabled eBee, it is possible to include RINEX-files (Receiver Independent Exchange Format) in the post-flight-processing in eMotion 3. If this function was not available, the reflectance maps were georeferenced manually using the Georeferencer Plugin in *QGIS* (QGIS Development Team, 2019). The extraction of the reflectance data of the sampling spots was undertaken in *R* (R Core Team, 2018) using the package *sf* (Pebesma, 2018), whereby all pixels of a plot were summarized as median.

Reflection values were transformed into *GAI* values using the vegetation index VIQUO (Bukowiecki et al., submitted manuscript). The linear model reaches a MAE of 0.44 m^2^ m^−2^ in an independent data set and showed to be stable during the whole growing season (applicability during senescence is demonstrated).

### Statistical Analysis

All data processing and statistical analysis were conducted in the statistical environment *R* (R Core Team, 2018), the package *ggplot2* was used for visualizations (Wickham, 2016).

A linear mixed model was used for the analysis of variance of the relationship of cultivar, crop management as well as year with all functional traits (*GY*, *BIO*, *HI*, *RI*, *RUE*):

(4) $$P_{\mathrm{ijklm}}=\infty +g_{i}+t_{j}+y_{k}+{\left(\mathrm{gt}\right)}_{\mathrm{ij}}+{\left(\mathrm{yt}\right)}_{\mathrm{kj}}\;+{\left(\mathrm{yg}\right)}_{\mathrm{ki}}+{\left(\mathrm{ytg}\right)}_{\mathrm{kji}}+B_{l}+{\left(\mathrm{BT}\right)}_{lj}+{\left(\mathrm{BTS}\right)}_{\mathrm{ljm}}+\varepsilon_{\mathrm{ijklm}},$$

where *P* *_ijklm_* is the phenotype of the *i*th cultivar, the *j*th crop management, the *k*th year, the *l*th block and the *m*th sub-block, *μ* is the general mean, *g* *_i_* is the fixed effect of the *i*th cultivar, *tj* is the fixed effect of the *j*th crop management, *y* *_k_* is the fixed effect of the *k*th year, (*gt*)*_ij_* is the fixed interaction of the *i*th cultivar in the *j*th crop management, (*yt*)*_kj_* is the fixed interaction of the *j*th crop management in the *k*th year, (*yg*)*_ki_* is the fixed interaction of the *i*th crop management in the *k*th year, (*ytg*)*_kji_* is the fixed interaction of the *i*th cultivar in the *j*th crop management and the *k*th year, *B* *_l_* is the random effect of the *l*th block, (*BT*)*_lj_* is the random interaction of the *j*th crop management in the *l*th block, (*BTS*)*_ljm_* is the random interaction of the *m*th sub-block in the *j*th crop management and the *l*th block, and *ε*_ijklm_ is the error term.

The model (and all subsequently described models with random components) was implemented with the package *lme4* (Douglas et al., 2015). Following Type II Wald chisquare test was conducted with the package *car* (John and Sanford, 2011).

For the estimation of variance components for each trait, a fully randomized model was used:

(5) $$P_{\mathrm{ijklm}}=\infty +G_{i}+T_{j}+Y_{k}+{\left(\mathrm{GT}\right)}_{\mathrm{ij}}+{\left(\mathrm{GY}\right)}_{\mathrm{ik}}+{\left(\mathrm{TY}\right)}_{\mathrm{jk}}\;+{\left(\mathrm{GT}Y\right)}_{\mathrm{ijk}}+{\left(\mathrm{YB}\right)}_{\mathrm{kl}}+{\left(\mathrm{YBT}\right)}_{\mathrm{kly}}+{\left(\mathrm{YBTS}\right)}_{\mathrm{klym}}+\varepsilon_{\mathrm{ijklm}},$$

where *P* *_ijklm_* is the phenotype of the *i*th cultivar, the *j*th crop management, the *k*th year, the *l*th block and the *m*th sub-block, *μ* is the general mean, *G* *_i_* is the random effect of the *i*th cultivar, *T* *_j_* is the random effect of the *j*th crop management, *Y* *_k_* is the random effect of the *k*th year, (*GT*)*_ij_* is the random interaction of the *i*th cultivar in the *jt*h crop management, (*GY*)*_ik_* is the random interaction of the *i*th cultivar in the *k*th year, (*TY*)*_jk_* is the random interaction of the *j*th crop management in the *k*th year, (*GTY*)*_ijk_* is the random interaction of the *i*th cultivar in the *j*th crop management and the *k*th year, (*YB*)*_kl_* is the random interaction of the *l*th block in the *k*th year, (*YBT*)*_klj_* is the random interaction of the *j*th crop management in the *l*th block and the *k*th year, (*YBTS*)*_kljm_* is the random interaction of the *m*th sub-block in the *j*th crop management and the *l*th block and the *k*th year, and *ε*_ijklm_ is the error term. The variances of (*YB*), (*YBT*) and (*YBTS*) are summed up to the component design.

Adjusted means for every cultivar in every year were calculated prior to the analysis of relationships between traits and year of variety release as well as the stepwise analysis of the contribution of traits to the yield formation to reduce the influence of soil properties and terrain. Because the experimental design (blocks, sub-blocks) did not ameliorate sufficiently, we added a continuous variable to the model which represents the effects of soil and terrain. The variable is calculated by the Papadakis-method (type PAP-8) described by Gezan et al. (2010). The final model for the calculation of adjusted means is:

(6) $$P_{\mathrm{ijkl}}=\mu +g_{i}+t_{j}+{\left(\mathrm{gt}\right)}_{\mathrm{ij}}+\text{soil}+B_{k}+{\left(\mathrm{TB}\right)}_{\mathrm{jk}}+{\left(\mathrm{STB}\right)}_{\mathrm{ljk}}+\varepsilon_{\mathrm{ijkl}},$$

where *P* *_ijkl_* is the phenotype of the *i*th cultivar, the *j*th crop management, the *k*th block and the *l*th sub-block, *μ* is the general mean, *g* *_i_* is the fixed effect of the *i*th cultivar, *t* *_j_* is the fixed effect of the *j*th crop management, (*gt*)*_ij_* is the fixed interaction of the *i*th cultivar in the *j*th crop management, *soil* is the continuous variable accounting for soil properties and terrain, *B* *_k_* is the random effect of the *k*th block, (*TB*)_jk_ is the random interaction of the *j*th treatment in the *k*th block, (*STB*)_ijk_ is the random interaction of the *l*th sub-block in the *j*th treatment and the *k*th block, and *ε*_ijkl_ is the error term.

Correlations between traits are calculated with the function *corr* in base *R* (R Core Team, 2018). The package *ggcorrplot* (Alboukadel, 2018) was used for visualizations. For the study of temporal changes, the same analysis is conducted with a 25-years sliding window over year of variety release creating multiple subsets of the dataset. Their results are assigned to the center of the considered period. The results of the first and final ten years are deleted to ensure a sufficient large subset.

The path analysis shows standardized beta coefficients (centered by mean and scaled by standard deviation) for relationships with inherent causal relationship (*GY* = *BIO* × *HI* and *BIO* = *RI* × *RUE*) and Pearson’s correlation coefficients for intercorrelations (*BIO* with *HI* and *RI* with *RUE*). The package *ggraph* is used for the visualization (Pedersen, 2019).

## Results

### Grain Yield, Biomass, and Harvest Index

**Figure 1:**
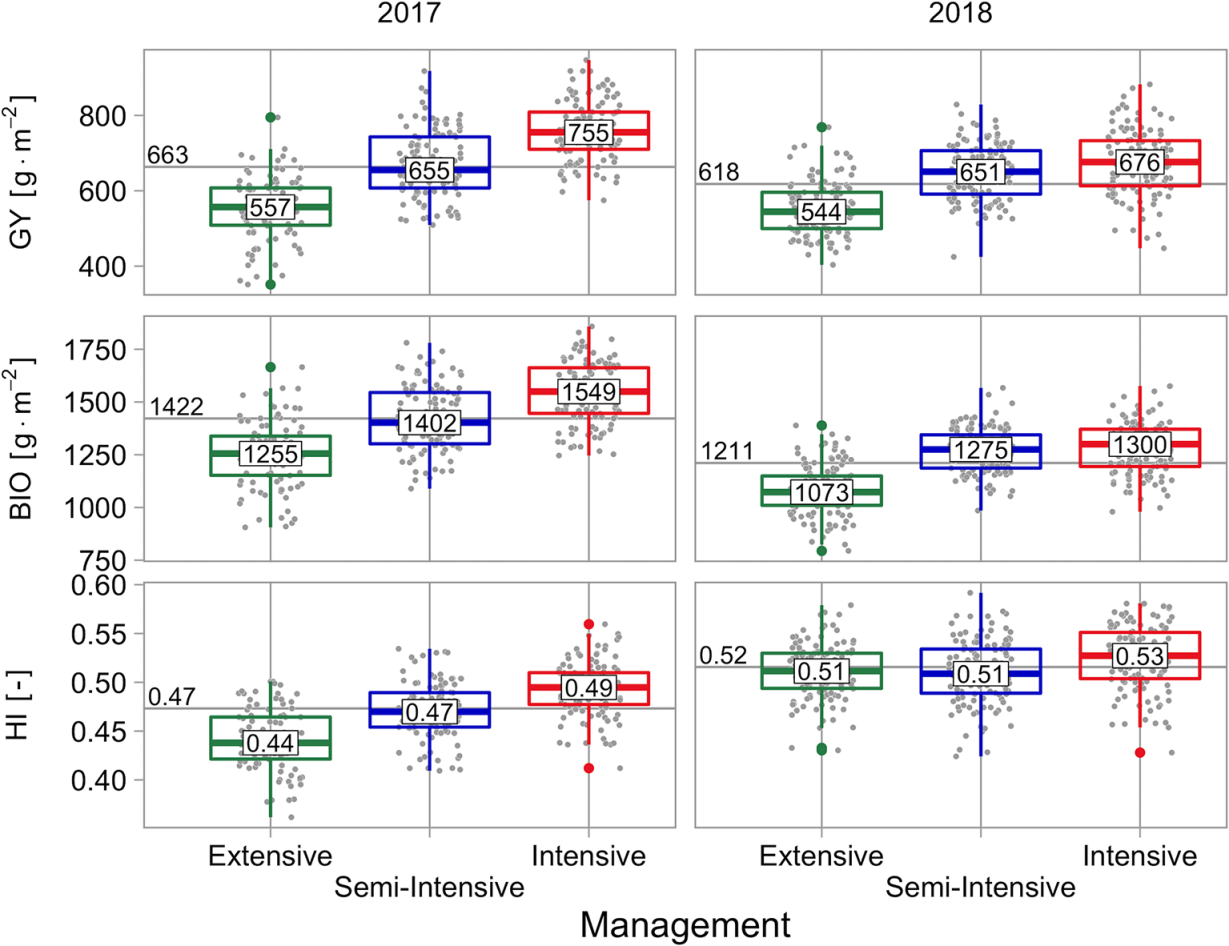
Grain yield (*GY*), final biomass (*BIO*) as well as harvest index (*HI*), grouped by management and experimental year. Values in boxplots reflect the median value of managements within experimental years, horizontal line and its label reflect the median value of the experimental year.

Grain yield (*GY*) ranged between 408 and 900 g m^−2^ over all cultivars, crop managements, and experimental years. Median values of crop managements raised from 553 g m^−2^ (extensive), over 653 g m^−2^ (semi-intensive) to 722 g m^−2^ (intensive). Median values of experimental years dropped from 663 g m^−2^ in the growing season 2016–2017 to 618 g m^−2^ in the growing season 2017–2018 (**Figure 1**).

There was a significant main effect of cultivar on *GY*, a significant main effect of crop management, and a significant main effect of experimental year. Additionally, there was a significant interaction effect between cultivar and crop management on *GY*, a significant interaction effect between cultivar and experimental year, and a significant interaction effect between cultivar, crop management, and experimental year. The interaction effect between crop management and experimental year was not significant (**Table 5**). The variance of *GY* was mainly explained by crop management, followed by cultivar. Experimental year is of exceptional low importance, almost only occurring in interaction with other predictors (**Figure 5**).

**Table 5:** Results of ANOVAs for all functional traits.

		DF	χ2	*p*
Grain yield	Cultivar	41	908.62	<.001
	Management	2	75.22	<.001
	Year	1	4.13	<.05
	Cultivar × Management	82	126.96	<.01
	Cultivar × Year	41	141.60	<.001
	Management × Year	2	5.7	.058
	Cultivar × Management × Year	74	108.05	<.01
Biomass	Cultivar	41	251.28	<.001
	Management	2	51.32	<.001
	Year	1	38.79	<.001
	Cultivar × Management	82	94.78	.16
	Cultivar × Year	41	123.41	<.001
	Management × Year	2	2.9	.23
	Cultivar × Management × Year	74	92.96	.067
Harvest index	Cultivar	41	1421.02	<.001
	Management	2	168.15	<.001
	Year	1	424.64	<.001
	Cultivar × Management	82	96.2	.14
	Cultivar × Year	41	184.17	<.001
	Management × Year	2	58.99	<.001
	Cultivar × Management × Year	74	91.45	.082
Radiation interception	Cultivar	41	322.11	<.001
	Management	2	80.14	<.001
	Year	1	21.11	<.001
	Cultivar × Management	82	110.05	<.05
	Cultivar × Year	41	108.18	<.001
	Management × Year	2	1.91	.38
	Cultivar × Management × Year	74	114.30	<.01
Radiation use efficiency	Cultivar	41	231.12	<.001
	Management	2	17.07	<.001
	Year	1	30.74	<.001
	Cultivar × Management	82	92.89	.19
	Cultivar × Year	41	103.82	<.001
	Management × Year	2	2.44	.29
	Cultivar × Management × Year	74	85.66	.17

The absolute drop of *GY* due to drought (*GY drop*) was higher for high *BIO* genotypes in the unstressed season 2017 (r = −0.48), correlations of *GY drop* and the subtraits of *BIO* as well as *GY* itself were consequently strong, too (*RUE:* r = −0.47; *RI:* r = −0.3; *GY:* r = −0.38). Genotypes with later ear emergence were stronger affected by drought, but correlations were very moderate (r = −0.11). *HI* was uncorrelated to *GY drop* (**Figure S4**).

Final biomass (*BIO*) ranged between 935 and 1707 g m^−2^ over all cultivars, crop managements, and experimental years. Median values of crop managements raised from 1131 g m^−2^ (extensive), over 1322 g m^−2^ (semi-intensive) to 1393 g m^−2^ (intensive). Median values of experimental years dropped from 1422 g m^−2^ in the 2016–2017 season to 1211 g m^−2^ in the 2017–2018 season (**Figure 1**).

There was a significant main effect of cultivar on *BIO*, a significant main effect of crop management, and a significant main effect of experimental year. Additionally, there was a significant interaction effect between cultivar and experimental year. The interaction effects between cultivar and crop management, between experimental year and crop management, and between experimental year, crop management, and cultivar were not significant (**Table 5**). The variance of *BIO* was nearly equally explained by crop management and experimental year, whereas cultivar had a quite low contribution (**Figure 5**).

The harvest index (*HI*) ranged between 0.38 and 0.57 over all cultivars, crop managements, and experimental years. Median values of crop managements were nearly unchanged from 0.49 (extensive), over 0.49 (semi-intensive) to 0.51 (intensive). Median values of experimental years showed an increase from 0.47 in the 2016–2017 season to 0.52 in the 2017–2018 season (**Figure 1**).

There was a significant main effect of cultivar on *HI*, a significant main effect of crop management, and a significant main effect of experimental year. Additionally, there was a significant interaction effect between cultivar and experimental year and a significant interaction effect between crop management and experimental year. The interaction effects between cultivar and crop management and between experimental year, crop management, and cultivar were not significant (**Table 5**). The variance of *HI* was mainly explained by experimental year, followed by cultivar. Crop management had a comparatively low contribution and the sum of design and residual term is exceptionally low, indicating a low influence by soil differences and/or low measurement errors (**Figure 5**).

### Radiation Interception and Radiation Use Efficiency

**Figure 2:**
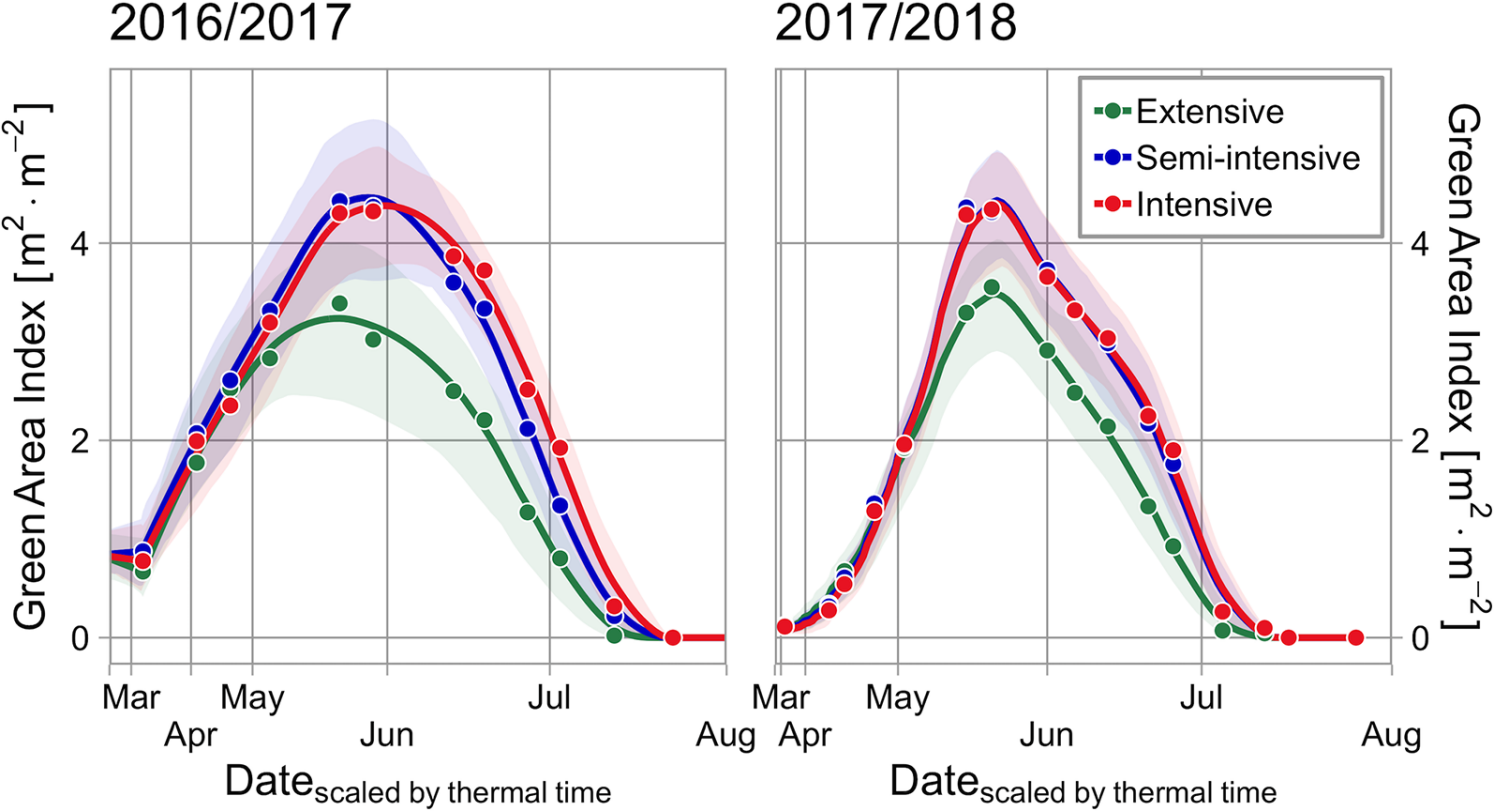
Comparison of *GAI* courses in the season 2016/2017 and 2017/2018. Lines and points represent the mean value of different managements, the pale ribbon indicates the range between the .05 quantile and .95 quantile.

Measured values of *GAI* showed a feasible course during both seasons and the applied interpolation method was an adequate compromise between smoothing and sufficient representation of the original data (**Figure 2**). The MAE between interpolated and original data was 0.1 m^2^ m^−2^ in the season 2016–2017 and 0.08 m^2^ m^−2^ in the season 2017–2018, respectively.

After late sowing in the season 2017–2018, values of *GAI* were much lower in early spring but the development of canopies caught up due to a rapid development in May and maximum *GAI* values were quite similar in both experimental years. In 2016–2017 the mean values of the whole cultivar set reached as its maximum peak value: 4.35 m^2^ m^−2^ (intensive), 4.43 m^2^ m^−2^ (semi-intensive), and 3.23 m^2^ m^−2^ (extensive). In the season 2017–2018 they reached: 4.38 m^2^ m^−2^ (intensive), 4.41 m^2^ m^−2^ (semi-intensive), and 3.47 m^2^ m^−2^ (extensive). In late May 2018, drought became severe and *GAI* values dropped rapidly followed by a fast senescence and early ripening (**Figure 2**).

**Figure 3:**
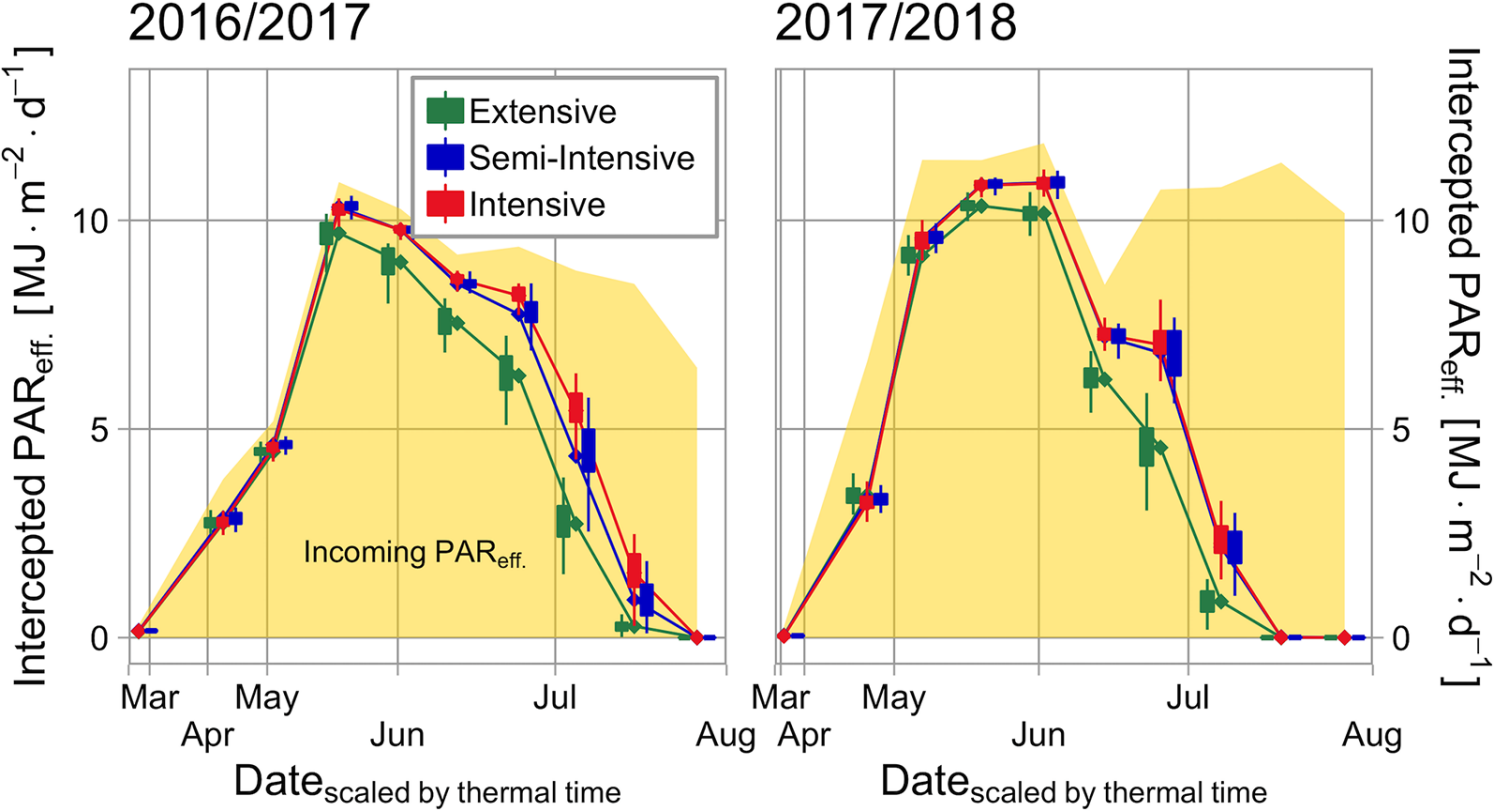
Seasonal course of the amount of intercepted effective radiation in the cultivar set and the incoming effective radiation (yellow area). Both seasons are segmented in 10 equidistant periods and their mean values are presented. Box-whisker-plots are dodged around the mean value (diamond shape), statistical outliers are excluded.

Canopies nearly reached total radiation interception when *GAI* reached its maximum, this was particular the case when abundant nitrogen is supplied (**Figure 3**). In the season 2016–2017 the mean value of the fraction of absorbed radiation over the whole cultivar set reached as its maximum peak value: 95% (intensive), 95% (semi-intensive), and 89% (extensive). In the season 2017–2018 they reached: 95% (intensive), 95% (semi-intensive), and 91% (extensive).

**Figure 4:**
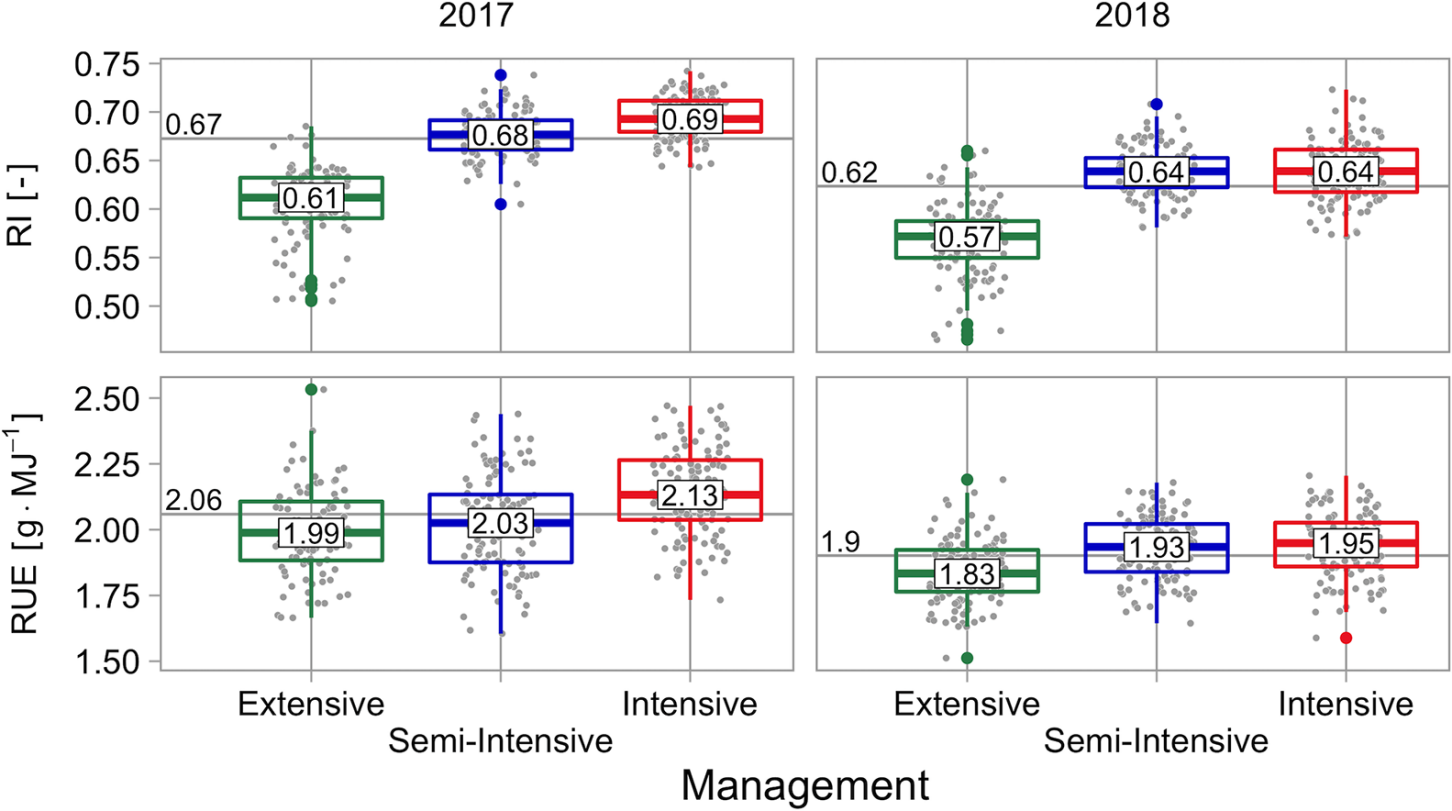
Fraction of intercepted radiation (*RI*) and radiation use efficiency (*RUE*), grouped by management and experimental year. Values in boxplots reflect the median value of managements within experimental years, horizontal line and its label reflect the median value of the year.

The fraction of intercepted radiation (*RI*) ranged between 0.51 and 0.72 over all cultivars, crop managements, and experimental years. Median values of crop managements raised from 0.59 (extensive), over 0.66 (semi-intensive) to 0.67 (intensive). Median values of experimental years dropped from 0.67 in the growing season 2016–2017 to 0.62 in the growing season 2017–2018 (**Figure 4**).

**Figure 5:**
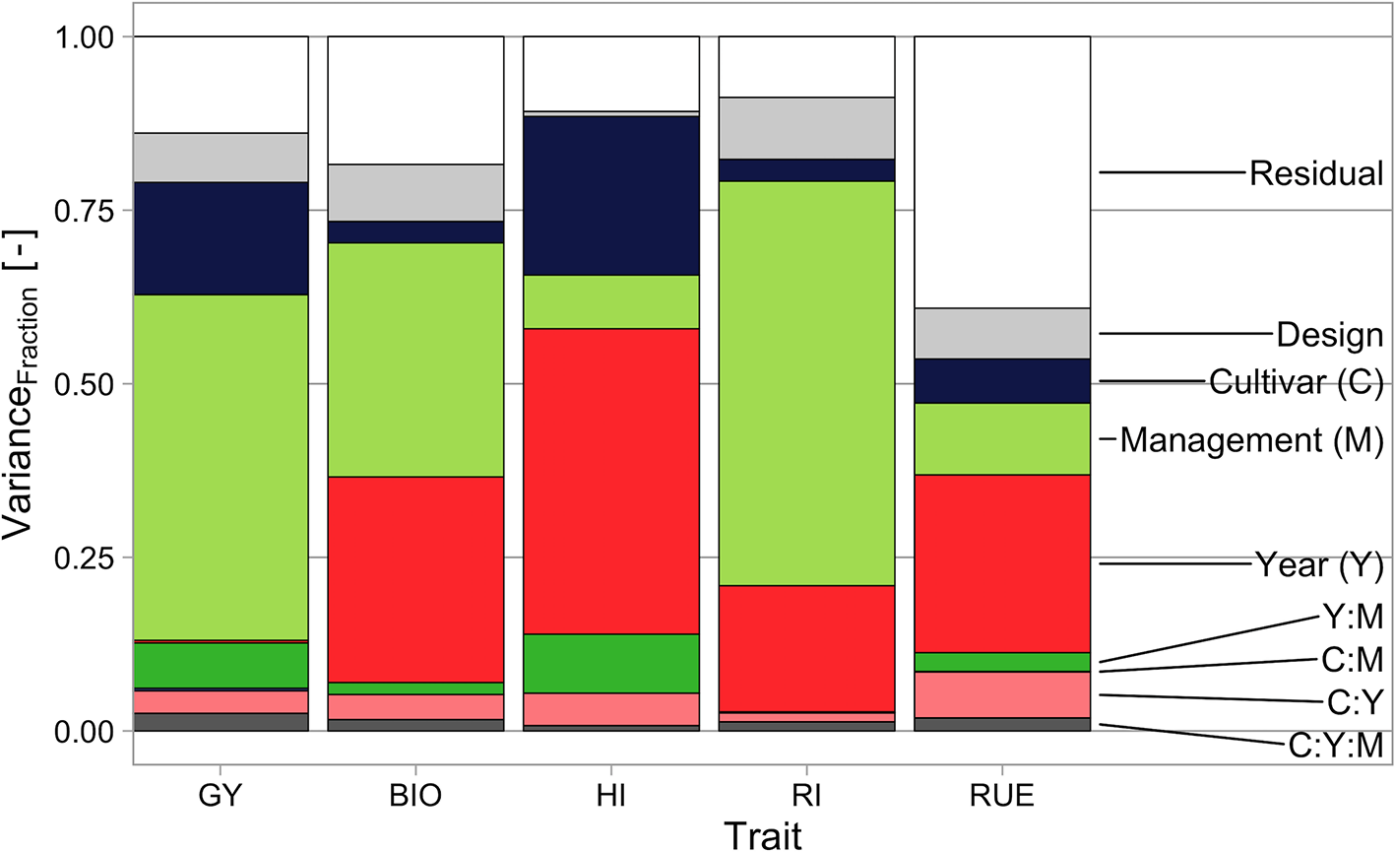
Fraction of variance explained by different variance components for the analyzed traits. *GY*, grain yield; *BIO*, final biomass; *HI*, harvest index; *RI*, fraction of intercepted radiation; *RUE*, radiation use efficiency.

There was a significant main effect of cultivar on *RI*, a significant main effect of crop management, and a significant main effect of experimental year. Additionally, there was a significant interaction effect between cultivar and crop management, a significant interaction effect between cultivar and experimental year, and a significant interaction effect between experimental year, crop management, and cultivar. The interaction effect between crop management and experimental year was not significant (**Table 5**). The variance of *RI* was mainly explained by crop management, followed by experimental year. Cultivar was of low importance, and interaction components are exceptionally low (**Figure 5**).

The radiation use efficiency (*RUE*) ranged between 1.68 and 2.33 g MJ^−1^ over all cultivars, crop managements, and experimental years. Median values of crop managements raised from 1.89 (extensive), over 1.96 (semi-intensive) to 2.03 g MJ^−1^ (intensive). Median values of experimental years dropped from 2.06 g MJ^−1^ in the growing season 2016–2017 to 1.90 g MJ^−1^ in the growing season 2017–2018 (**Figure 4**).

There was a significant main effect of cultivar on *RUE*, a significant main effect of crop management, and a significant main effect of experimental year. Additionally, there was a significant interaction effect between cultivar and experimental year. The interaction effect between cultivar and crop management, between crop management and experimental year, and between cultivar, crop management, and experimental year were not significant (**Table 5**). The variance of *RUE* was mainly explained by experimental year, followed by crop management and cultivar. The sum of design and residual term was exceptionally high, indicating a high influence by soil differences and/or high measurement errors, maybe as a result of blown up errors because the calculation is based upon biomass and intercepted radiation (**Figure 5**).

### Breeding Progress

To evaluate the breeding progress related to functional traits, relationships between traits and year of variety release were analyzed. The linear regression models of all traits within year and crop management showed positive slopes and the vast majority of them were significant. Non-significant slopes mainly occurred within the trait *RI* (**Table 6**). The scatterplot of this trait indicates that here, progress and regress alternated during breeding history (**Figure S2**). Progress of the highest-order trait *GY* as the mean of both years was 3.4 g m^−2^ y^−1^ in the intensive crop management, was slightly lower in the extensive management with 3.2 g m^−2^ y^−1^, and was highest in the semi-intensive management with 4.0 g m^−2^ y^−1^. *GY* progress as the mean of all managements within years dropped from 3.8 g m^−2^ y^−1^ in the season 2016–2017 to 3.2 g m^−2^ y^−1^ in the season 2017–2018. Relationships between ear emergence and year of variety release were not significant.

**Table 6:** Relationship between functional traits and year of variety release.

Trait	Year	Management	R^2^	P value	Slope
Grain yield [g m^−2^]	2017	Extensive	0.58	5.10 e −09 (***)	3.9
		Semi-intensive	0.48	3.40 e −07 (***)	4.2
		Intensive	0.47	4.60 e −07 (***)	3.3
	2018	Extensive	0.47	4.80 e −07 (***)	2.4
		Semi-intensive	0.64	2.40 e −10 (***)	3.7
		Intensive	0.54	3.00 e −08 (***)	3.5
Biomass [g m^−2^]	2017	Extensive	0.27	3.80 e −04 (***)	4.2
		Semi-intensive	0.30	1.60 e −04 (***)	5.0
		Intensive	0.10	0.038 (*)	2.3
	2018	Extensive	0.29	2.10 e −04 (***)	2.4
		Semi-intensive	0.44	1.60 e -06 (***)	3.3
		Intensive	0.32	1.00 e −04 (***)	3.0
Harvest index [–]	2017	Extensive	0.57	7.80 e −09 (***)	0.0016
		Semi-intensive	0.43	2.20 e −06 (***)	0.0013
		Intensive	0.50	1.70 e −07 (***)	0.0013
	2018	Extensive	0.37	1.70 e −05 (***)	0.0011
		Semi-intensive	0.51	1.20 e −07 (***)	0.0015
		Intensive	0.51	1.10 e −07 (***)	0.0015
Intercepted radiation [MJ m^−2^]	2017	Extensive	0.18	0.0055 (**)	8.50 e −04
		Semi-intensive	0.07	0.086 (ns)	3.90 e −04
		Intensive	0.04	0.18 (ns)	2.80 e −04
	2018	Extensive	0.03	0.31 (ns)	2.20 e −04
		Semi-intensive	0.20	0.0031 (**)	5.40 e −04
		Intensive	0.06	0.1 (ns)	3.20 e −04
Radiation use efficiency [g MJ^−1^]	2017	Extensive	0.20	0.0028 (**)	0.0037
		Semi-intensive	0.32	9.40 e −05 (***)	0.0061
		Intensive	0.08	0.062 (ns)	0.0023
	2018	Extensive	0.31	1.30 e −04 (***)	0.0032
		Semi-intensive	0.36	2.50 e −05 (***)	0.0034
		Intensive	0.31	1.40 e −04 (***)	0.0035
Ear emergence [°C]	2017	Intensive	0.08	0.066 (ns)	–0.63
	2018	Intensive	0.05	0.17 (ns)	–0.40

R2, P value, and slope of the linear model trait explained by year of release are shown for every trait, year, and management combination. Comments in parenthesis: P value >= 0.05 (ns), P value < 0.05 (*), P value < 0.01 (**), P value < 0.001 (***).

The coefficient of determination was highest for the trait *GY*, with an R^2^ value of 0.53 as the mean of all management year combinations and decreased over *HI* (R^2^: 0.48), *BIO* (R^2^: 0.29), and *RUE* (R^2^: 0.26) to *RI* (R^2^: 0.10) (**Table 6**).

**Figure 6:**
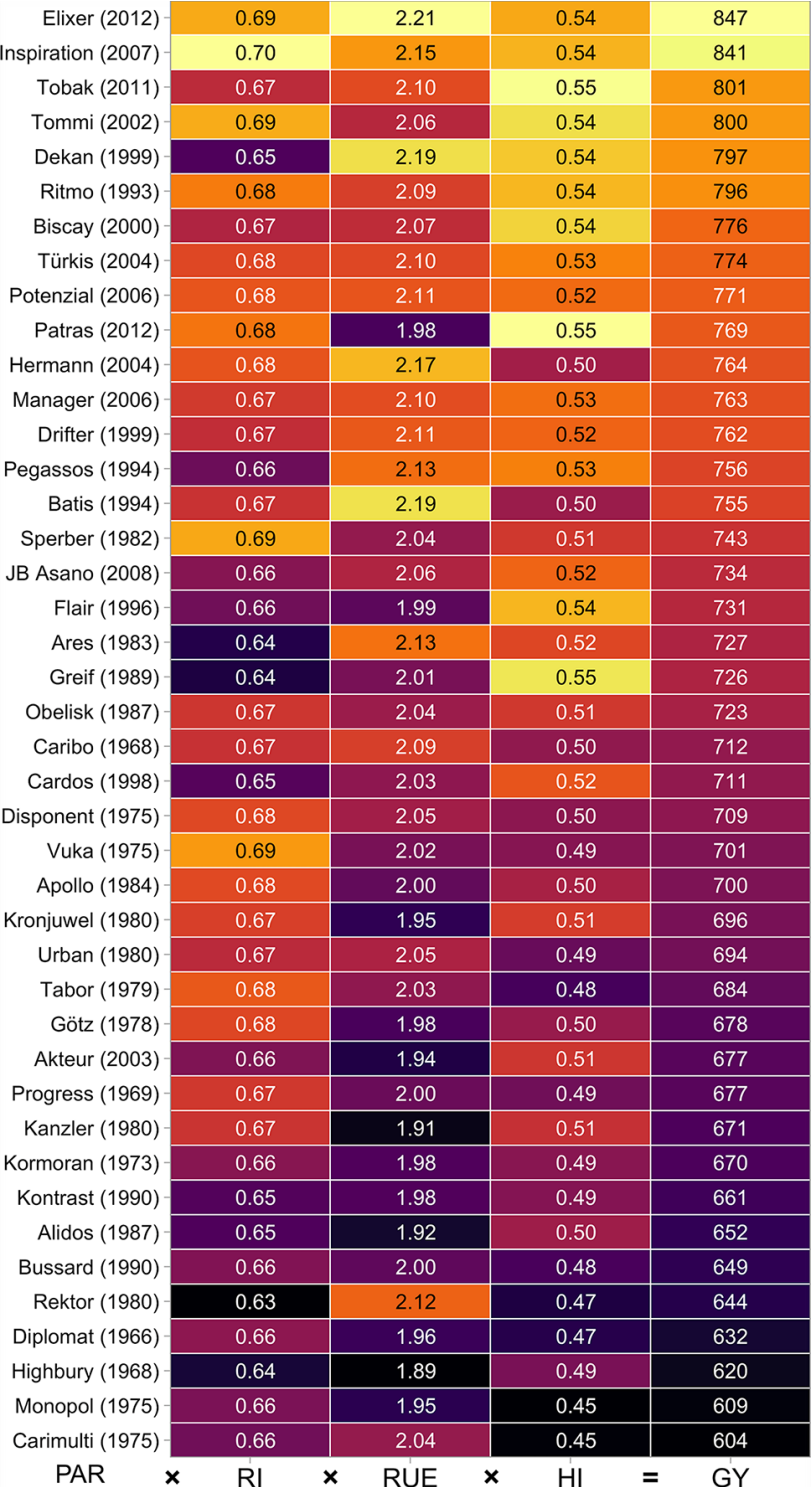
Combination of functional traits in the cultivar set. Mean values of the intensive crop management over both experimental years are shown. Values in parentheses specify the year of release.

This decline of the coefficient of determination—going down from grain yield to the sub- and sub-sub-traits—is reflected in a qualitative view on the cultivar set. The straight increase of grain yield was accompanied by a quite vague increase of its functional sub-traits and similar yields can be reached by quite different pathways (**Figure 6**).

Besides the analysis of temporal changes of functional traits, an analysis of their importance to the formation of their higher-order traits (*GY* = *BIO* × *HI*, *BIO* = *RI* × *RUE*) is of interest. Even a trait without any temporal trend can be of high importance for the explanation of differences between cultivars.

**Figure 7:**
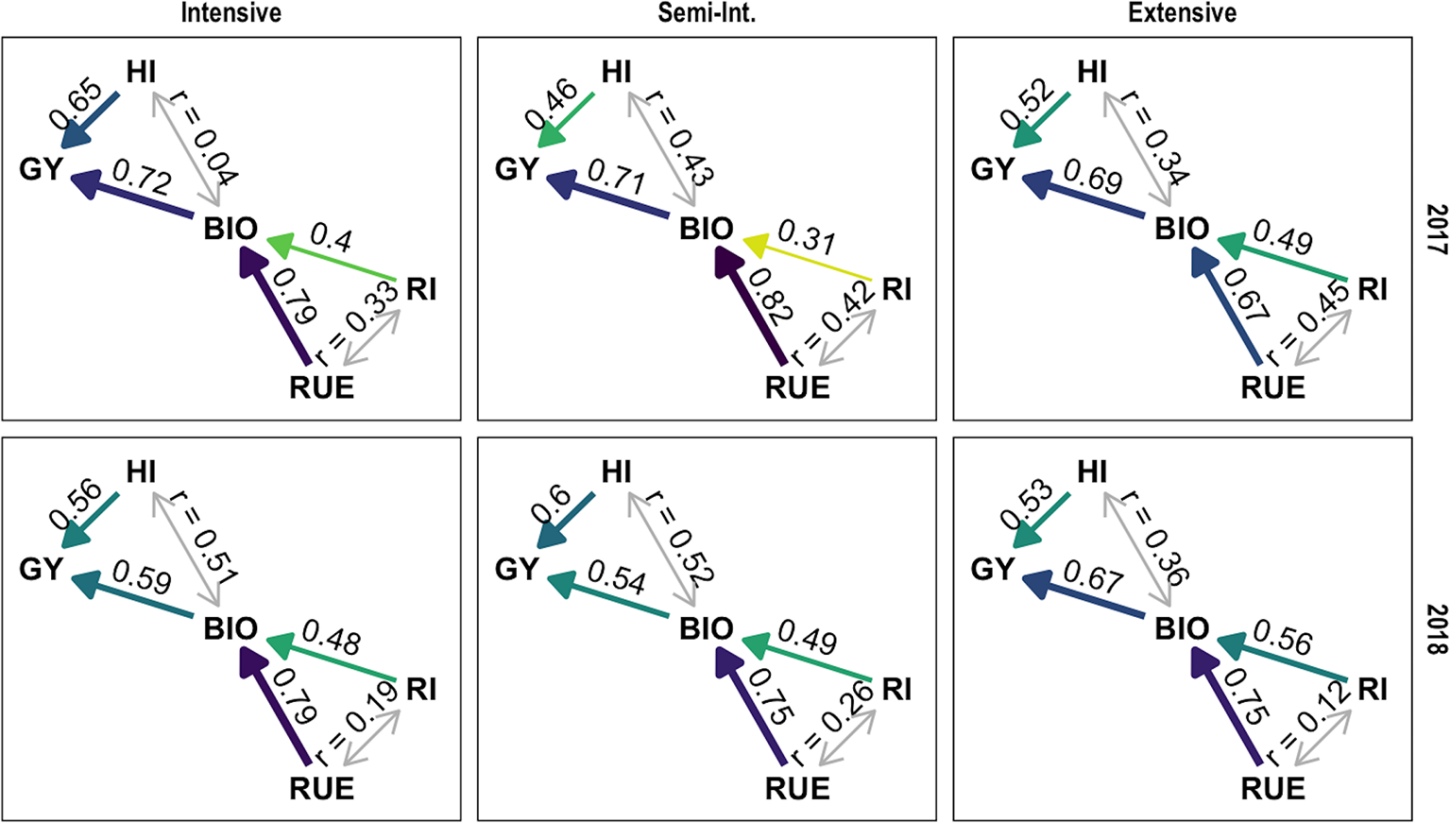
Path analysis of functional traits during breeding history, grouped by growing seasons and crop management. Implicitly causal relationships show standardized beta coefficients (one headed arrow) other relationships show Pearson’s correlation coefficient (two headed arrow).

Standardized beta coefficients of the linear model *GY* = *BIO* × *HI* were higher for *BIO* in the growing season 2016–2017, in the growing season 2017–2018 *HI* and *BIO* were roughly of equal importance, except in the extensive crop management where the relationship was nearly unchanged. Both traits were nearly uncorrelated in the intensive crop management during the first season but moderate to strong correlated in all other management year combinations (**Figure 7**).

Standardized beta coefficients of the linear model *BIO* = *RI* × *RUE* were higher for *RUE* in all management year combinations and the coefficient of *RI* was especially low in the semi-intensive management during the first season, indicating that infections primarily affected radiation use efficiency. Semi-intensive and intensive crop management behaved quite similar in the second season which reflects the low infection pressure in the second season. Coefficient of *RI* was comparatively high in the extensive crop management. Both traits were moderately correlated in the 2017 season and only weak correlated in the 2018 season (**Figure 7**).

A broader view of correlations between all functional traits showed that no negative intercorrelations existed. Except the correlation between *HI* and *BIO* (as well as *RI* and *RUE*) in the intensive crop management and the non-drought experimental year, these positive correlations were surprisingly high. In the season 2017, later ear emergence was moderately positive correlated to *RI* and *BIO* and moderately negative correlated to *HI*. In the season 2018, ear emergence was nearly uncorrelated to all other traits (**Figure S3**).

**Figure 8:**
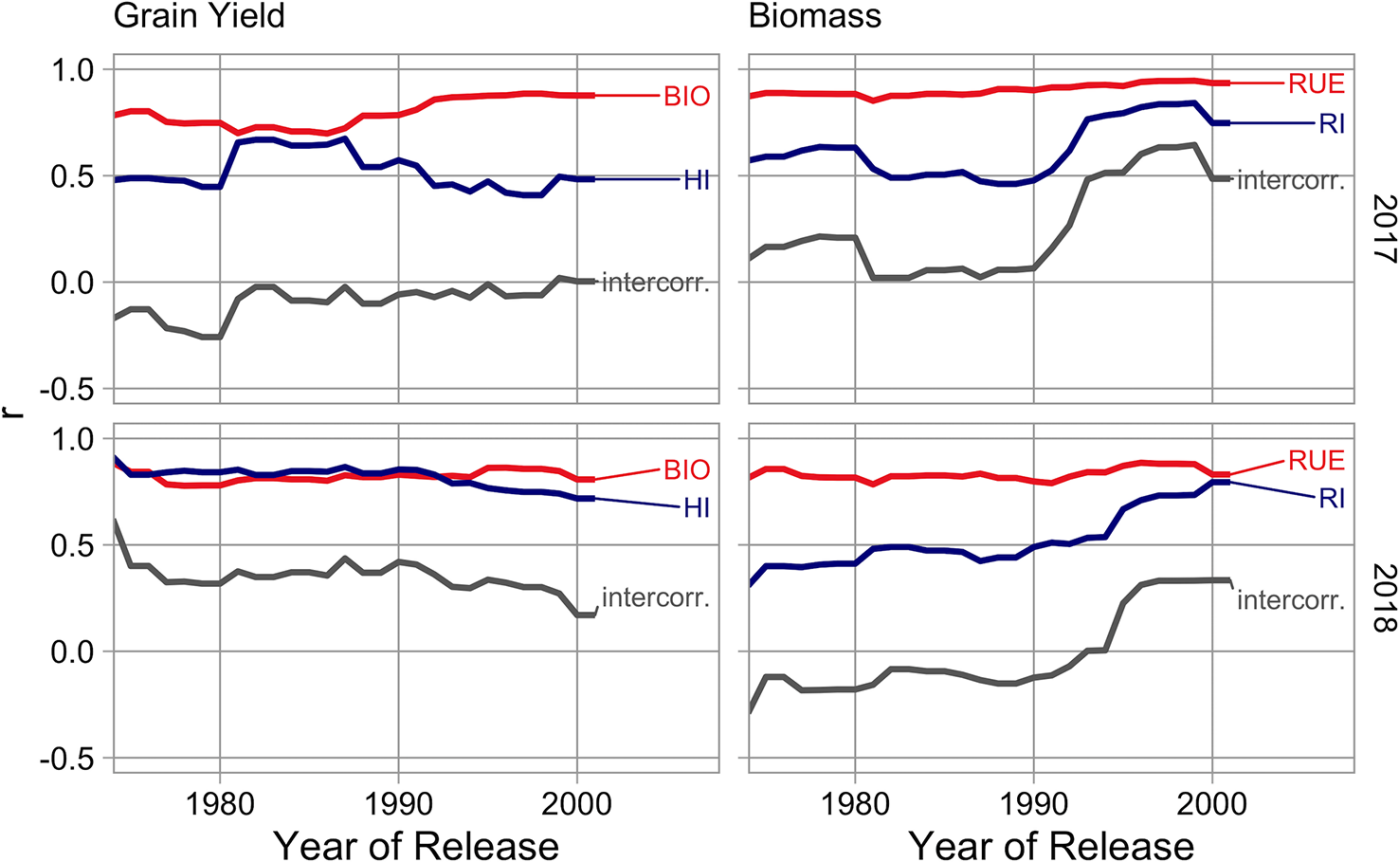
Temporal changes of the contribution of traits during breeding history in different growing seasons in the intensive crop management. Left: Correlation between grain yield (*GY*) and harvest index (*HI*) as well as biomass (*BIO*). Right: Correlation between biomass (*BIO*) and radiation interception (*RI*) as well as radiation use efficiency (*RUE*). The component intercorr. is the correlation between explanatory variables.

The preceding static view is complemented with a dynamic view in the intensive crop management. The importance of *BIO* outpaces the importance of *HI* around the year 1990 (**Figure 8**, left). On the contrary, the distance between *RUE* and *RI* diminished in the 90s. Interestingly, correlation between both traits increased appreciable at the same time (**Figure 8**, right).

## Discussion

### Grain Yield, Harvest Index, and Biomass

In contrast to the yield stagnation observed in the agricultural practices (Lin and Huybers, 2012), *GY*, as the mean value of all management year combinations, increased with 3.5 g m^−2^ y^−1^ between 1966 and 2012 and the data clearly showed that this progress continues. Yield progress was usually higher when growing conditions were less optimal like it has been shown by Voss-Fels et al. (2019). The extensive crop management in the experimental year 2018 (drought) with an increase of 2.4 g m^−2^ y^−1^ was an exception. Less mineralization might have resulted in very low N supply which could have been a resource limitation for higher yields (**Table 6**). All growing conditions included, *GY* of more recent cultivars was always higher than that of older ones. This absolute yield under diverse growing conditions—unlike some definitions of yield stability—is the most relevant measure regarding food security (Snowdon et al., 2019).

*GY* is mainly influenced by crop management, followed by cultivar and experimental year (mainly occurring in interaction with crop management) (**Figure 5**). The effect of drought in 2018 might be underestimated due to both suboptimal crop managements. Here, the effects are biased due to differing infection pressure and the uniform application of growth regulators in the second experimental year that shifted the *HI* of the extensive crop management considerably upwards (**Figure 1**). The yield drop of 10% in the intensive crop management represents best the observed effect of drought. Notably, the biomass drop of 16% was partly compensated by an increase in *HI* (**Figure 1**).

High biomass genotypes in the unstressed season (2017) were stronger affected by drought, reflecting the tight coupling of biomass production and transpiration. On the contrary, *HI* is a resource neutral trait and was uncorrelated to *GY drop*. Earlier ear emergence had a small positive influence on the effect of drought (**Figure S4**).

*HI* showed, with a mean R^2^ of 0.48 of all relationships to year of variety release, the most directional development during breeding history of all subtraits (**Table 6**). This might have two reasons: it is the most considered subtrait by breeders and it is resource neutral. Additionally, until it eventually reaches some physiological boundaries (Berry et al., 2007), it might have very little negative interactions with other subtraits.

Maximum values of *HI* reached 0.55 (**Figure 6**), a value slightly higher than previously reported ones: 0.46 (Brancourt-Hulmel et al., 2003), 0.50 (Schittenhelm et al., 2019), 0.52 (Rose et al., 2017), and 0.53 (Shearman et al., 2005). They slowly approach the theoretical thresholds, hypothesized by Austin (1980) (0.62), Foulkes et al. (2010) (0.64), and Shearman et al. (2005) (0.66).

The contribution of *BIO* and *HI* to the breeding progress of *GY* is a frequently researched question. Many studies observe an exclusive explanation of the progress regarding *GY* by an increase in *HI* (Austin, 1980; Austin et al., 1989; Siddique et al., 1989; Slafer and Andrade, 1989; Brancourt-Hulmel et al., 2003; Acreche et al., 2008; Tian et al., 2011; Zheng et al., 2011), other studies show a contribution of both traits (Hucl and Baker, 1987; Donmez et al., 2001; Shearman et al., 2005; Sanchez-Garcia et al., 2013; Beche et al., 2014; Gaju et al., 2016), and only a few studies detect no influence of *HI* (Waddington et al., 1986; Silva et al., 2014).

We observed a slightly higher correlation between *GY* and *BIO* than with *HI*. The contribution of *HI* was increased in the second experimental year with drought (**Figure 7**) and was variable in the breeding history (**Figure 8**). This dependency on environment and cultivar set corresponds to the variability of results in the literature. The contribution of *HI* decreased in recent breeding history (**Figure 8**) and latest cultivars consistently reach values above 0.5 in the intensive crop management (**Figure S2**). It seems like best cultivars recently converge to theoretical maximum values and potential for improvements are slowly diminishing but the trait should not be regarded as settled. *HI* and *BIO* were nearly uncorrelated in the intensive crop management during the first season but moderately to highly correlated in all other management year combinations (**Figure 7**), indicating that all types of stress are mainly affecting grain filling and consequently both traits in parallel.

*HI* was moderately negatively correlated to ear emergence (**Figure S3**), so early flowering was one component of high *HI* values, but a big part of variation was independent of it.

### Radiation Interception and Radiation Use Efficiency

The challenge of an adequate mapping of *GAI* values during the whole season was implemented with a multispectral approach proposed by Bukowiecki et al. (submitted manuscript). The expansion of the application of spectral reflection measurements to the phase of senescence with vegetation indices, like the NDVI, has been shown before (Lopes and Reynolds, 2012). We extended this approach with a more functional perspective—going from the measurement of *GAI* to the calculation of *RI*. The resulting courses during senescence (**Figure 2**) are in agreement with multiple authors who interpolated this phase by some logistic-alike model with an accelerating and decelerating phase (Pepler et al., 2005; Christopher et al., 2008; Vijayalakshmi et al., 2010; Christopher et al., 2014; Christopher et al., 2016; Luo et al., 2018).

Due to nearly complete interception during full canopy development, differences in the cultivar set regarding *RI* mainly occurred in early and late season (**Figure 3**). These phases correspond to the key words “early vigor” and “stay green.” High genetic variability for these traits has been shown before: “early vigor” (Turner and Nicolas, 1998; Rebetzke et al., 2001; Rebetzke et al., 2004; Maydup et al., 2013; Zhang et al., 2014), “stay-green” (Joshi et al., 2007). In comparison, “stay green” was of much higher importance because here, cultivar differences coincide with high incoming radiation. Both traits differentiated more in the season 2017–2018. This might be linked to late establishment intensified by high incoming radiation in April and May (“early vigor”) (Moore and Rebetzke, 2015) and drought (“stay green”) (Idso et al., 1980; Christopher et al., 2008; Graziani et al., 2014; Thomas and Ougham, 2014; Christopher et al., 2016; Christopher et al., 2018). Under reduced nitrogen supply (extensive crop management), appreciable differences of *RI* do occur in mid-season, too (**Figure 3**). Here, maximum *GAI* values are on a level where genotypic differences regarding *GAI* do result in noticeable differences for radiation interception (exponential term of Beer-Lambert law). Consequently, the importance of radiation interception for the explanation of final biomass is highest in the extensive crop management (**Figure 7**).

Like Kitonyo et al. (2017) have shown for delayed senescence, we did not detect a linear trend of radiation interception in most management year combinations with the year of variety release (**Table 6**). It seems like progress and regress alternated during breeding history regarding this trait (**Figure S2**).

We were not able to differentiate between cosmetic and functional “stay-greens” which is in general difficult in large field trials (Rebetzke et al., 2016) but a multitude of authors have shown a linking between chlorophyll content and net photosynthetic rate during senescence (Luo et al., 2006; Waters et al., 2009; Derkx et al., 2012; Naruoka et al., 2012). This indicates that the functional type of “stay green” is rather the rule than the exception in wheat. Nonetheless, occurring non parallel progression of *GAI* and photosynthesis might result in low values of *RUE*, as discussed later.

*RUE* ranged between 1.68 and 2.33 g MJ^−1^ over all cultivars, crop managements, and experimental years and was highly affected by all occurring forms of stress—drought, nitrogen deficiency, and pests. The effect of drought is reflected in the drop of −0.15 g MJ^−1^ from the experimental year 2017 (non-drought) to the year 2018 (drought), the effect of nitrogen deficiency in the drop of −0.1 g MJ^−1^ from the semi-intensive to the extensive crop management in the experimental year 2018 (very low infection pressure), and the effect of pests in the drop of −0.1 g MJ^−1^ from the intensive to the semi-intensive crop management in the experimental year 2017 (high infection pressure). In comparison to management and experimental year, the variance component of cultivars is low but a quite high and significant interaction between cultivar and experimental year occurred (**Figure 5**).

The observed values are lower than reported ranges by Shearman et al. (2005) between 2.33 to 2.64 g MJ^−1^, Sadras et al. (2012) between 1.54 to 2.68 g MJ^−1^ and the range from 2.29 to 2.57 g MJ^−1^ we observed in a preceding trial (Rose et al., 2017). The lower end of the range is highly depending on the minimum year of release of the cultivar set and the amount of occurring stresses, but the upper end should be on a similar level.

The calculation of *RUE* requires an adequate description of radiation interception, so most authors (and the mentioned studies) confine themselves to the pre-anthesis phase. Due to a new calibration of a multispectral sensor (Bukowiecki et al., submitted manuscript) we were able to expand the calculation until harvest. We hypothesize that the *RUE* during senescence is reduced due to the degradation of rubisco and assume that this is the reason for comparably low values.

*RUE* showed a significant linear increase during breeding history except in the intensive crop management during the season 2016–2017 (non-drought) (**Table 6**). Slopes range between 0.0023 and 0.0061 g MJ^−1^ per year and are much lower than reported values by Sadras et al. (2012), 0.012 g MJ^−1^ per year. In their Mediterranean environment, evapotranspiration is much higher than rain during the growing season. These results indicate that historical trends of *RUE* should not only be seen in the context of photosynthesis but in the context of the possibilities of genotypes to cope with adverse growing conditions (drought, nitrogen deficiency, pests). Additionally, Sadras et al. (2012) showed the link of *RUE* to canopy traits instead of photosynthesis per se. Here, a better light distribution leads to an increased canopy photosynthesis (Zhu et al., 2010).

In comparison, *RUE* is of higher importance for the explanation of *BIO* than *RI* but nearly equal levels are reached in the extensive crop management (**Figure 7**). A more detailed analysis of temporal changes in the intensive crop management showed that *RI* was clearly less important for most of the breeding history but became nearly equally important during the nineties (**Figure 8**). Both traits are positively correlated which might express that beside the phenological component of “stay green” it also just reflects the ability to cope with the external factors: drought, nitrogen depletion, and fungal infections (Richards, 2000; Rebetzke et al., 2016). Additionally, results of Zhang et al. (2006) suggest, that prolonged retention of high chlorophyll concentrations can be in certain cases an indirect indicator for higher levels of rubisco in previous phases, instead of the direct cause of increased light absorption. A lower intercorrelation in the experimental year 2018, with occurring drought, might reflect a negative relationship between water use (high radiation interception) and water supply in a later phase (low radiation use efficiency). *RI* was moderately correlated to ear emergence (**Figure S3**), so later flowering was one component of high *RI* values, but a big part of variation was independent of it.

### Breeding Progress—Past and Future

Our results show that the stagnation of wheat yields in Central-Europe, is not accompanied by an ending of breeding progress. The coefficient of determination of the linear relationships with year of variety release was highest for the trait *GY*, with an R^2^ value of 0.53 as the mean of all management year combinations and decreased over *HI* (R^2^: 0.48), *BIO* (R^2^: 0.29), and *RUE* (R^2^: 0.26) to *RI* (R^2^: 0.10). This reflects the main procedure in breeding—a selection for higher *GY*. The functional background is usually unknown and progress in *GY* is often accompanied by regress in some of its sub-traits (**Figure 6**). The combination of best performers in the sub-traits: *HI*, *RUE*, and *RI* for crosses is an appealing approach but negative relationships between these traits should exist. Conversely, all traits in the analyzed cultivar set are positively correlated (**Figure S3**) but it must be kept in mind that nearly 50 years of indirect parallel selection for better *HI*, *RUE*, and *RI* constitute the genetic material. Phenotyping of lower level functional traits (*RI*, *RUE*, *HI*) in combination with genotyping would help to detect QTLs which might be assignable to some quite stable trait (e.g. leaf angle, specific leaf area, initiation of senescence). This might help to understand the complex interaction of genotype and environment.

Time of ear emergence was nearly unchanged during the breeding period (**Table 6**) and showed little variation (**Table 1**) which reflects quite strict conceptions of optimal flowering time by local farmers and the consideration of this requirement by breeders.

A frequently manifested hypothesis is that plant breeding brought *HI* and *RI* close to their theoretical maxima and only *RUE*, determined by photosynthesis, is left for improvements, e.g. Long et al. (2006). Our results, representing breeding history in Central-Europe, only partly agree with this assumption. The contribution of *HI* for the explanation of *GY* diminishes since around 1990 (**Figure 8**) but modern cultivars do not finally reach theoretical maximum values (**Figure 6**). So, progress might still be possible, but a lower rate has to be expected. Regarding *RI*, in contrast to the mentioned hypothesis improvements continue and are becoming more important in recent years (**Figure 8**). Slopes of linear relationships between *RI* and year of variety release are in most management year combinations not significant (**Table 6**) and some high yielding cultivars express low values in this trait (**Figure 6**). This indicates, not a level around a theoretical maximum, but exploitable variation. As a restriction it has to be stated, that high *RI* genotypes may be more affected by drought stress and late ripening cultivars are not always accepted by farmers, especially in humid regions as the risk of high grain moisture contents at harvest increases.

The progress in the development of small unmanned drones, in combination with calibrated spectral sensors allows comparable low-cost high-throughput phenotyping of this trait. Due to its close correlation with final biomass, especially under suboptimal nitrogen supply (**Figure 7**), *RI* might serve as a proxy for biological performance in early breeding generations when yield cannot be reasonable ascertained.

As we discussed earlier, the stagnation of wheat yields in Central-Europe is not caused by a lack of breeding progress-so other reasons have to be responsible. The analysis of variance components shows that the impact of crop management and experimental year (mainly water supply) on *GY* and its sub-traits was much higher than that of cultivar (**Figure 5**). The reasons for yield stagnation might belong in this sphere.

## Conclusion

Our results show that the stagnation of wheat yields in Central-Europe, is not accompanied by a lack of breeding progress. This equally applies to suboptimal growing conditions like restricted pesticide applications and limited water- or nitrogen-supply. Besides the ongoing increase in grain yield (*GY*), nearly all sub-traits showed a parallel development, but relationships are weaker on the lower levels of yield formation. One exception of this steady breeding progress is radiation interception (*RI*), here, phases of progress and regress alternate. Differences in the cultivar set mainly occur in the phase of senescence. In combination with the strong contribution of *RI* to biomass production (*BIO*), the nondirectional development in the past indicates some exploitable potential for breeders. Additionally, we have demonstrated that the trait can be measured with a non-destructive high-throughput approach. Biomass itself is getting more important in comparison to harvest index (*HI*). Values of harvest index are slowly approaching theoretical maxima and correlations with grain yield are decreasing.

The detailed analysis of yield formation reveals that high-yielding cultivars often underperform in some sub-traits. A better knowledge of these functional traits during the breeding process might help to enable an even straighter yield progress.

## Data Availability Statement

The datasets generated for this study are available on request to the corresponding author.

## Author Contributions

TR was responsible for the data acquisition in the field and all data processing as well as data analysis. HK contributed by the formulation of the main research ideas and accompanied the process of manuscript writing.

## Funding

This work was supported by the Federal Ministry of Education and Research (BMBF) (grant number: 031A354D).

## Conflict of Interest

The authors declare that the research was conducted in the absence of any commercial or financial relationships that could be construed as a potential conflict of interest.
