# Supplementary material for: The Contribution of Functional Traits to the Breeding Progress of Central-European Winter Wheat Under Differing Crop Management Intensities
Source: Front Plant Sci. 2019 Dec 6;10:1521. doi: 10.3389/fpls.2019.01521 (PMC6908521; doi:10.3389/fpls.2019.01521)
Supplement: Supplementary file 1 [file Presentation_1.pdf]

Supplementary Material

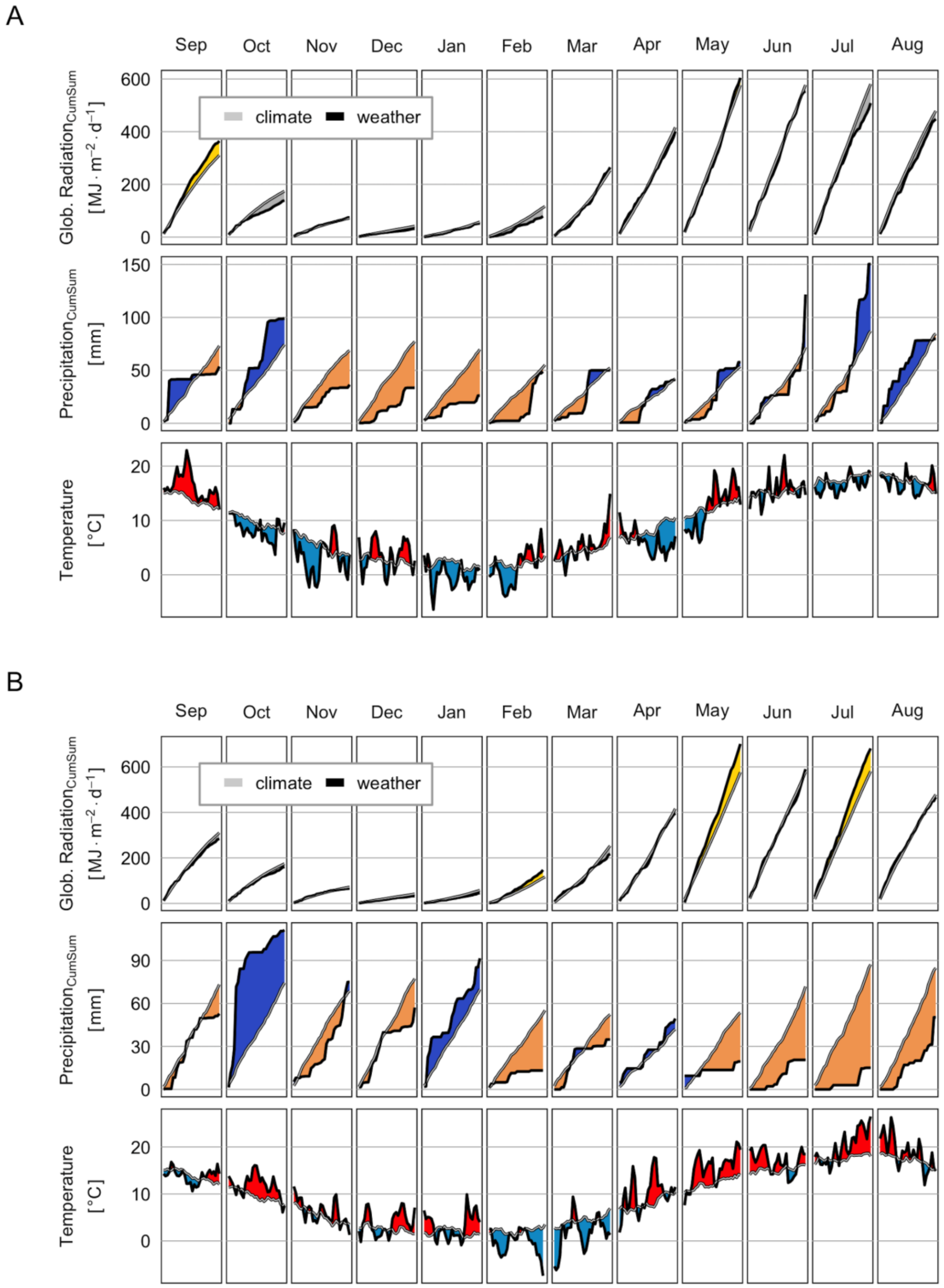

Supplementary Figure 1. Weather in comparison to climate during the growing seasons 2016–2017 (A) and 2017–2018 (B). Weather below and above the longtime average (30 years) are separated by different colors of the areas.

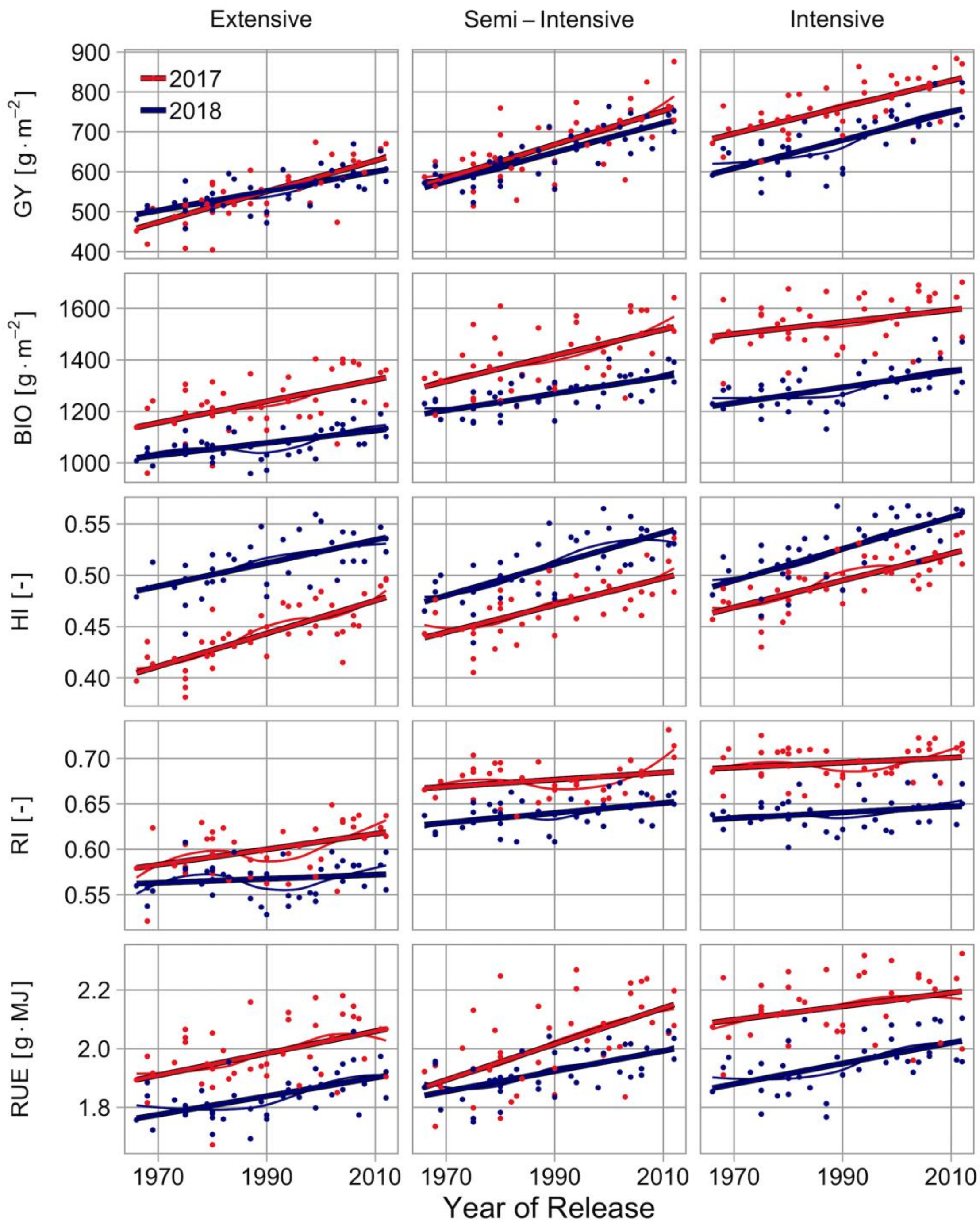

Supplementary Figure 2. Relationship between functional traits and year of variety release, grouped by crop management and growing season. The bold line with black border shows a linear fit between trait and year of release. The narrow line shows a LOESS-smoother (α = 0.8) to get a picture of non-linear behavior. Traits are: grain yield (*GY*) with 100 % DM, biomass (*BIO*) with 100 % DM, harvest index (*HI*), fraction of intercepted radiation (*RI*), and radiation use efficiency (*RUE*).

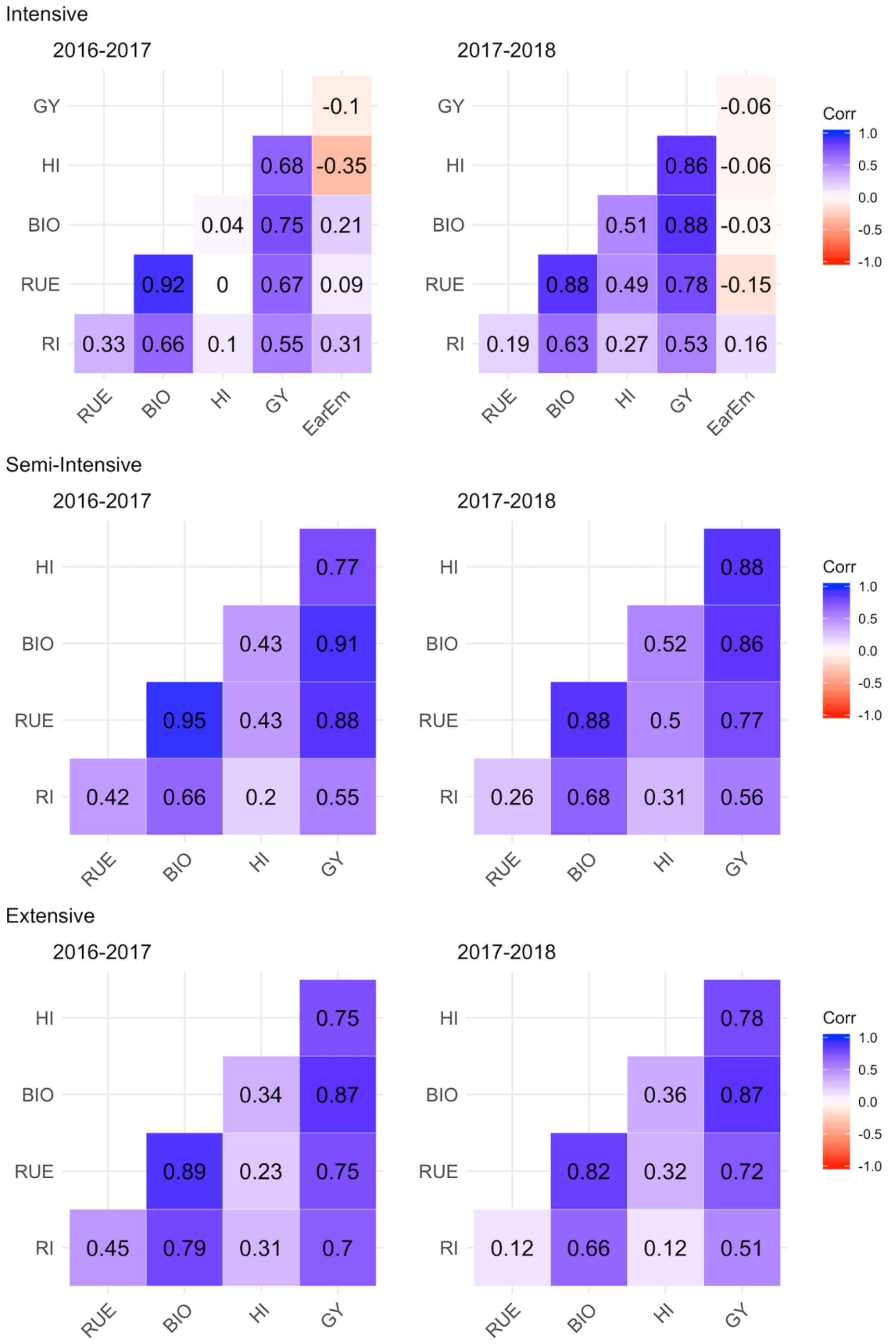

Supplementary Figure 3. Correlation between all functional traits and ear emergence grouped by experimental year and crop management. Traits are: grain yield (*GY*) with 100 % DM, biomass (*BIO*) with 100 % DM, harvest index (*HI*), fraction of intercepted radiation (*RI*), radiation use efficiency (*RUE*), and ear emergence (*EarEm*). Ear emergence was only measured in the intensive crop management.

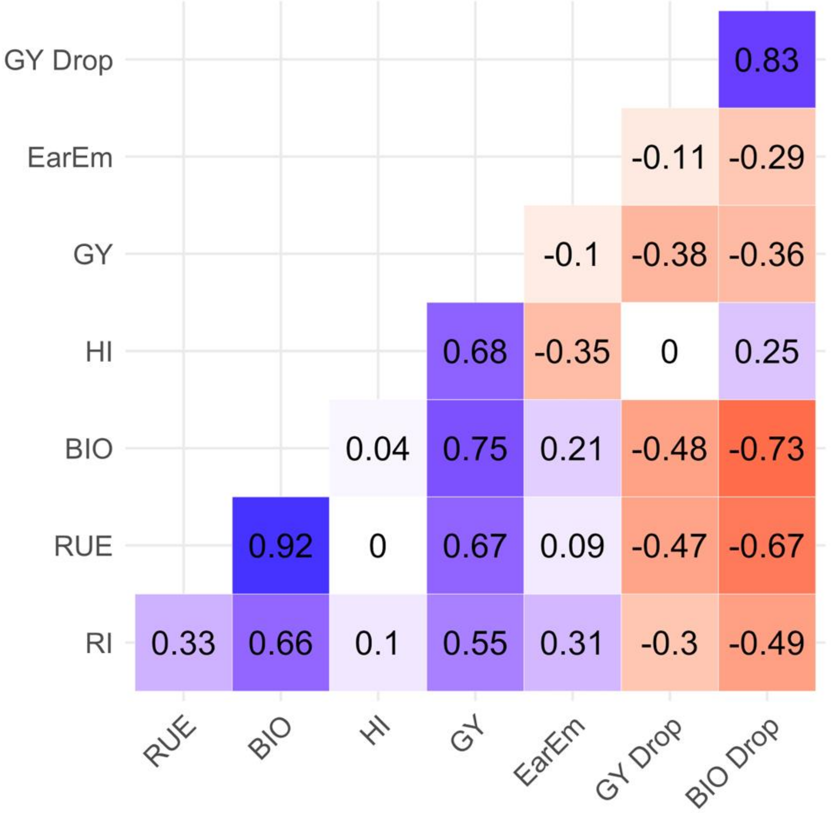

Supplementary Figure 4. Correlation between all functional traits and ear emergence in the 2017 season (non-drought) and the drop of grain yield/biomass in the 2018 season (drought). Dataset is restricted to the intensive crop management. Traits are: grain yield (*GY*) with 100 % DM, biomass (*BIO*) with 100 % DM, harvest index (*HI*), fraction of intercepted radiation (*RI*), radiation use efficiency (*RUE*), and ear emergence (*EarEm*), drop of grain yield (*GY Drop*) and drop of biomass (*BIO Drop*). The lower the values for drop traits (usually more negative) the more severe is the effect of drought.
